# A 45-Year-Old Farmer with an Ulcerative Rash, Shock, and Hemorrhagic Meningitis

**DOI:** 10.4269/ajtmh.2011.11-0238

**Published:** 2011-11-01

**Authors:** Mario Ponce, Alberto Mendoza, Carlos Seas

**Affiliations:** Instituto de Medicina Tropical Alexander von Humboldt, Universidad Peruana Cayetano Heredia, Lima, Peru; Instituto Nacional de Salud, Lima, Peru; Departamento de Enfermedades Infecciosas y Tropicales, Hospital Nacional Cayetano Heredia, Lima, Peru

**Figure 1.**
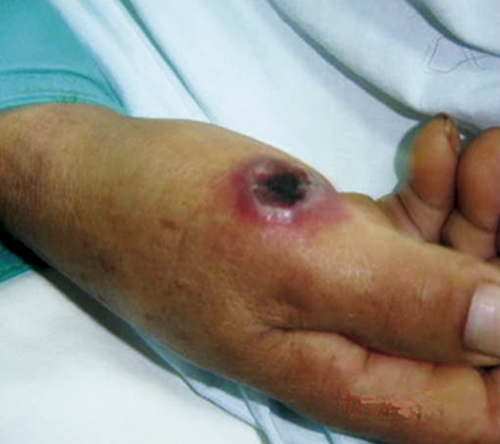
Ulcerative skin lesion located on the right hand. Note the black eschar at the bottom and the non-pitting edema surrounding the lesion.

**Figure 2.**
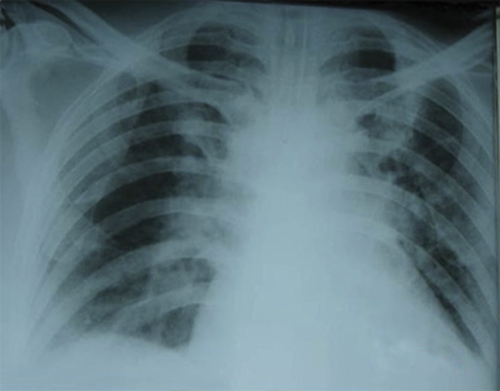
Chest x-ray showing bilateral alveolar infiltrates, predominantly in the right lung and bilateral pleural effusion. Note the marked widening of the mediastinum.

A 45-year-old male farmer was admitted with a 5-day history of fever, retrosternal chest pain, and mental obtundation. He lived in the highlands of Peru and had been in contact with the skin and viscera of deceased cattle for unknown reasons 8 days before initiation of symptoms. The physical examination showed fever, shock, a painless ulcerative skin lesion on the right hand (Figure 1), and meningeal signs. The chest x-ray showed bilateral pleural effusion, diffuse alveolar infiltrates, and a widened mediastinum (Figure 2). *Bacillus anthracis* was isolated from an ulcer sample and from the cerebrospinal fluid (CSF), which disclosed hemorrhagic features. Human anthrax results from direct inoculation through the skin, ingestion, or inhalation of spores. Inhaled spores are transported to the mediastinal lymph nodes, where they germinate and induce extensive necrosis with further hematogenous spread to other organs. The recommended antibiotic treatment for anthrax meningitis is a combination of a fluoroquinolone plus one or two additional agents with good CSF penetration (penicillin or ampicillin; meropenem; rifampicin; vancomycin).^1^ Inhalational anthrax carries a 92% mortality rate; meningoencephalitis is almost always lethal.^2^ Our patient had both cutaneous and inhalational anthrax and died despite all medical efforts.
